# Research of Dynamic Tensile Properties of Five Rocks under Three Loading Modes Based on SHPB Device

**DOI:** 10.3390/ma15238473

**Published:** 2022-11-28

**Authors:** Diyuan Li, Jinyin Ma, Quanqi Zhu, Bang Li

**Affiliations:** School of Resources and Safety Engineering, Central South University, Changsha 410083, China

**Keywords:** dynamic Brazilian splitting, digital image correlation, rock materials, SHPB

## Abstract

The validity of calculating the dynamic tensile strength of rock materials based on dynamic Brazilian tests is problematic. In order to gain a deeper understanding of the effects of three typical loading methods on the damage mechanism of rock specimens in the dynamic Brazilian tests, five different rocks were selected for the study. In the constant incident energy dynamic Brazilian test, the loading modes had a significant effect on the loading rate and dynamic tensile strength of the specimen, with the highest loading rate and tensile strength of the specimens under mode-III loading, followed by mode-I loading and mode-II loading. A high-speed camera and the digital image correlation (DIC) technique were used to successfully capture the rupture process of the Brazilian disc during impact loading. The evolution of the displacement and strain fields of the specimen was obtained by DIC technique, and four typical failure patterns and two rupture characteristics in the dynamic Brazilian test were summarized. The loading mode determined the crack initiation position of the specimen in the dynamic Brazilian test. The results showed that the mode-III loading is the most consistent with the Brazilian test theory, while the mode-II loading violates the test principle.

## 1. Introduction

Rock masses in deep underground projects are usually subjected to dynamic loads caused by excavation, blasting, and drilling [1,2,3,4,5,6,7,8,9,10]. Since local tensile failure often occurs under dynamic loads, many scholars have been attracted to conduct research on the dynamic tensile properties and fracture mechanism of rock materials [11,12,13,14]. The test methods for rock tensile strength include direct stretching and indirect stretching, i.e., the Brazilian disc [15,16]. The dynamic Brazilian test based on the split Hopkinson pressure bar (SHPB) is a popular and commonly used method to measure the indirect tensile strength of rock materials [17,18]. Rose et al. [19] were the first to use SHPB system to conduct dynamic Brazilian tests on concrete specimens and obtain the dynamic tensile strength of concrete. In addition, the dynamic tensile strength of ceramics was also measured by Johnstone et al. [20]. Subsequently, scholars have carried out a large number of dynamic Brazilian tests on rock materials. Based on a series of static and dynamic Brazilian tests, Gomez et al. [21] studied the influence of damage degree on tensile strength of concrete and granite. Dai et al. [22] explored several fundamental issues in dynamic Brazilian tests through experimental and numerical studies, such as the dynamic stress balance, the validity of the static standard equation, and the necessity of the arc loading method in dynamic Brazilian tests. Zhang et al. [23] investigated the dynamic mechanical properties and strain field evolution of marble specimens using combination of digital image correlation (DIC) technique and a high-speed camera in the dynamic Brazilian tests. In addition, the reasons for the discrepancy between the results of the dynamic Brazilian test and the direct tensile test were explained based on experimental observation and micromechanical models [24]. Wu et al. [14] investigated the accuracy of the unidirectional strain calculation method in the dynamic Brazilian test using two types of disc specimens based on the theory of stress wave propagation at the interface and verified the reasonableness of the results through tests. Moreover, different factors have been considered in the dynamic Brazilian tests to carry out relevant studies, such as temperature [18], anisotropy [25], and water saturation [26].

In fact, the validity of the dynamic Brazilian test is also problematic. Local crushing failure may occur at the end of the disk under impact loads, which is contrary to the hypothesis of the Brazilian test. Related scholars show different views on this. Wang et al. [27] proposed the flattened Brazilian test, that was, cutting a pair of loading surfaces parallel to each other at both ends of the disc, which could prevent premature failure caused by high stress concentration at the contact end of the specimen and the elastic bar. When the flattened loading angle is greater than 20°, the crack initiation position is in the center of the disk. Dai et al. [22] pointed out through an experimental study that the use of arc loading was not necessary in dynamic Brazilian tests of rocks, since the presence or absence of an arc loading device did not have a large effect on the dynamic tensile strength. Gomez et al. [21] added a pair of liners on the contact surface between the specimen and the elastic bars to prevent the localized failure at the end of the disc caused by point load in the impact test. In addition, some improvements were made to the loading device with the aim of improving the stress condition between the specimen and loading platens, including the addition of curved loading jaws.

Due to the limitations of indoor tests, some test parameters and test conditions were sometimes not satisfied. With the development of computer technology and related theories, many scholars have conducted rich research on dynamic Brazilian tests based on different numerical simulation methods. Ruiz et al. [28] used the 3D (three dimensional) finite element method (FEM) to simulate dynamic Brazilian tests on concrete materials and found that the dynamic strength of concrete was rate-sensitive, with an increasing strain rate. Zhu et al. [29] used the FEM analysis method (RFPA) to study the static and dynamic damage mechanisms of rock disks in Brazilian tests. Mahabadi et al. [30] developed a coupled finite element and discrete element method (FEM/DEM) to research the mechanical behavior of Barre granite based on the Brazilian tests, and the results matched well with the experimental results. In addition, Zhu et al. [29] and Ruiz et al. [28] verified the reliability of quasi-static standard computational equations in dynamic Brazilian tests based on numerical tests.

Throughout the above literature, the current studies on dynamic Brazilian tests are mainly on the dynamic strength and damage modes of specimens under conventional loading methods. Few literatures have systematically investigated the effects of different loading methods on the dynamic mechanical properties and damage behavior of Brazilian specimens. Thus, three different loading methods and five types of rocks were considered, and the deformation and damage patterns of each specimen were studied by using a high-speed camera and the DIC technique in order to obtain more comprehensive results. The key findings of this work are essential for the accurate use of the Brazilian test to obtain tensile strength and other information on rock specimens.

## 2. Experimental Procedure

### 2.1. Specimen Preparation

**Figure 1:**
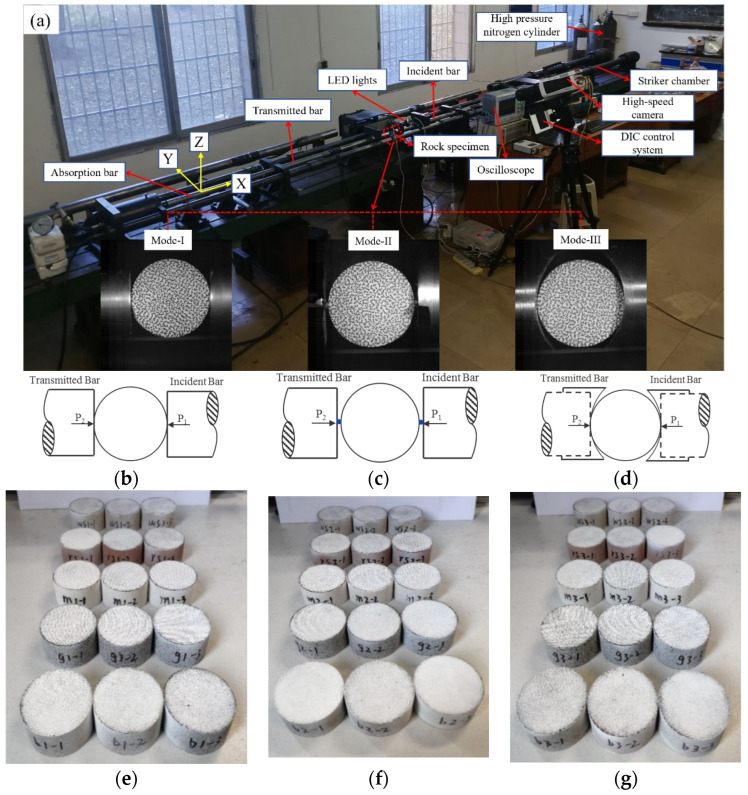
(**a**) Diagram of experimental device for the dynamic Brazilian tests and the high-speed photography system; the diagram for platform loading (**b**), steel bar loading (**c**), and arc loading (**d**); photographic views of the specimen under mode-I loading (**e**), mode-II loading (**f**), and mode-III loading (**g**).

Five typical rock types are selected in this paper, including white sandstone (WS), red sandstone (RS), marble (M), granite (G), and basalt (B), which are consistent with our previous study [31,32]. Three Brazilian disc specimens, with the dimensions of 50 mm × 25 mm (diameter × thickness), were prepared for each group of tests due to three different loading modes. Therefore, nine specimens were prepared for each lithology for a total of 45 specimens, as shown in Figure 1e–g. The dimensions of each specimen were measured, as shown in Table 1, and all specimens met the test requirements. According to the method recommended by the International Society for Rock Mechanics (ISRM) [33], the surfaces of the disc were ground by the diamond grinding wheel so that the parallel error of the two ends was less than 0.25° and the flatness error was less than 0.25 mm. These specimens for each lithology were taken from intact rocks blocks, respectively, without obvious fractures or bedding planes. Before the impact test is carried out, an artificial random speckle should be made on the specimen surface first due to the application of DIC technology and the lack of natural contrast in rocks.

According to the principle of DIC technique, a good speckle pattern usually meets the following conditions: (1) high-contrast; (2) consistent speckle sizes; (3) random distribution; (4) 50% coverage [34,35,36,37]. The speckle pattern was made on the specimen surface by means of plate printing and spray painting [38,39]. A printing plate is specially designed to print black spots with diameter of 0.66 mm on the surface of the specimen. First, a brush was used to clean the stains on the specimen surface, and then the white matte paint was used to spray on it to form a thin and uniform primer. After the white paint was solidified, the surface sprayed with white paint was vertically pressed 3–5 times on the printing plate to form random speckles. For each lithology, three standard specimens of 100 mm × 50 mm (height × diameter) were prepared for static uniaxial compression tests. The average uniaxial compressive strength, Young’s modulus, and Poisson’s ratio of five rock materials were obtained, as shown in Table 2. In Table 2, “*σ_c_*”, “*E*”, and “*ν*” represent the uniaxial compressive strength, Young’s modulus, and Poisson’s ratio, respectively. The specimen number was recorded as A-B-C, in which A represents the rock type, B represents the loading mode, and C represents the serial number of the specimens.

### 2.2. Experimental Setup and Testing Method

#### 2.2.1. SHPB System

**Figure 2:**
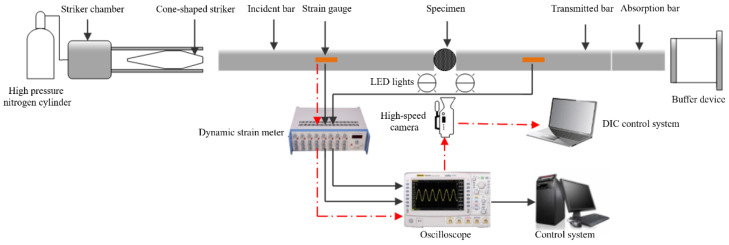
Schematic diagram of experimental device for dynamic Brazilian test and the high-speed photography system.

The dynamic Brazilian test using the split Hopkinson pressure bar (SHPB) system is the ISRM recommended method for determining the dynamic tensile strength of rock materials [40]. The schematic diagram of test principle based on the improved SHPB device and DIC technique is shown in Figure 2. The main components of the SHPB system are three elastic bars with a diameter of 50 mm: the incident bar, the transmitted bar, and the absorption bar. The three elastic bars and the cone-shaped striker were made of 40 Cr alloy with a density of 7817 kg/m^3^, an elastic modulus of 233 GPa, and a longitudinal wave velocity of 5458 m/s. In addition, the experimental device also includes a high-pressure nitrogen cylinder, a striker chamber, a dynamic strain meter, an oscilloscope, a high-speed camera, a buffer device, two LED (light-emitting diode) lights, and two control systems. Once the experimental device and specimen were ready, the cone-shaped striker was then ejected from a high-pressure chamber and impacted the free end of the incident bar. A compression wave began to propagate in the incident bar (incident wave, *ε_I_*). When propagating to the interface between the specimen and the incident bar, part of the wave is reflected back to the incident bar (reflected wave, *ε_R_*), and the rest of the wave passes through the specimen to the transmitted bar (transmitted wave, *ε_T_*). The signals of these three stress waves are recorded by the strain gauge bonded on bars, in which the strain gauge on the incident bar records the incident and reflected waves, while the strain gauge on the transmitted bar only records transmitted waves.

Based on one-dimensional stress wave theory, the dynamic pressure on the incident end (*P*_1_) and transmitted end (*P*_2_) of the specimen are [41]:

(1) $$P_{1}=A_{b}E_{b}\left(\varepsilon_{I}+\varepsilon_{R}\right)$$

(2) $$P_{2}=A_{b}E_{b}\varepsilon_{T}$$

The velocities at the contact end face between the incident bar (*v*_1_), transmitted bar (*v*_2_), and the specimen are:

(3) $$v_{1}=C_{b}\left(\varepsilon_{I}-\varepsilon_{R}\right)$$

(4) $$v_{2}=C_{b}\varepsilon_{T}$$

Therefore, the displacement of the specimen at the ends of the incident bar (*u*_1_) and transmitted bar (*u*_2_) can be obtained as follows:

(5) $$u_{1}=C_{b}\int_{0}^{t}\left(\varepsilon_{I}-\varepsilon_{R}\right)dt$$

(6) $$u_{2}=C_{b}\int_{0}^{t}\varepsilon_{T}dt$$

In Equations (1)–(6), *E_b_*, *A_b_*, and *C_b_* are the elastic modulus, the cross-sectional area, and the one-dimensional longitudinal stress wave velocity of the elastic bar, respectively; *ε_I_*, *ε_R_*, and *ε_T_* are the incident strain signals, reflected strain signals, and transmitted strain signals, respectively.

Similar to the principle of the static Brazilian test method, the dynamic Brazilian test is based on the same fact that the tensile strength of rock is much lower than the compressive strength. Therefore, when the Brazilian disk is subjected to radial load, the specimen will be failure somewhere in the center due to the tensile stress distributed along the diameter of the loading direction. Assuming that stress balance is satisfied at both ends of the specimen in the dynamic Brazilian test, the dynamic tensile strength (*σ_td_*) can be determined by the following formula [24]:

(7) $$\sigma_{td}=\frac{2P_{fd}}{\pi Dt}$$

where *P_fd_* is the dynamic peak load of the specimen; *D* and *t* are the diameter and thickness of the specimen. According to the Brazilian test theory, the application of this formula is also only applicable to the test of crack initiation in the central region of the disc. The tensile strength values calculated by Equation (7) in this paper are mainly used for comparison under different loading modes.

#### 2.2.2. Loading Mode

As shown in Figure 1, three loading modes are adopted, including platform loading (mode-I), steel bar loading (mode-II), and arc loading (mode-III). Under platform loading, the incident bar and transmitted bar in SHPB system were used to clamp the specimens directly. Two small steel bars made of the same material as the incident bar are fixed at the end of the incident bar and transmitted bar. The dynamic Brazilian test under mode-II were carried out by clamping the specimen with two steel bars. In addition, in order to realize the dynamic Brazilian test of mode-III loading, a pair of arc-shaped loading molds were designed and machined, whose materials were consistent with the incident bar and transmitted bar.

#### 2.2.3. High-Speed Camera and Digital Image Correlation System

A high-speed camera and digital image correlation techniques were used to monitor the deformation and failure characteristics of specimen surface in dynamic Brazilian tests. As shown in Figure 1, the lens of the high-speed camera was perpendicular to the specimen surface, and the resolution of the image was set to 256 × 256 pixels. The image was captured at a frame rate of 79,166 fps (i.e., an inter-frame time of 12.6 μs). In order to achieve the synchronous control of high-speed camera and SHPB system, the external trigger end of the high-speed camera was connected with oscilloscope, and the TTL (transistor transistor logic) signal generated by oscilloscope when recording the stress wave signal of the incident bar would synchronously trigger the high-speed camera. The digital image correlation system is a non-contact technique for identifying the distribution and evolution of strain or displacement. Compared with the traditional strain or displacement measurement method (e.g., strain gauge, extensometer), DIC has many advantages such as non-contact, high reliability, and full-field measurement [42,43,44,45]. In this research, the images collected in dynamic Brazilian tests were imported into two-dimensional visual image correlation software (VIC-2D) to obtain the evolution law of the strain and displacement fields on the disk surface.

## 3. Experimental Results and Discussion

### 3.1. Dynamic Stress Equilibrium

**Figure 3:**
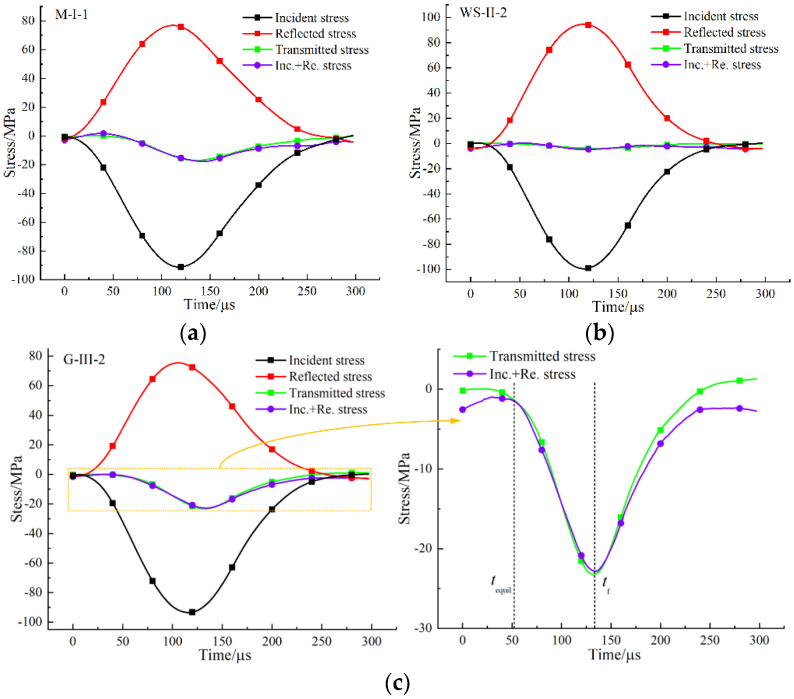
Dynamic stress history of typical specimens under different loading modes: (**a**) M-I-1; (**b**) WS-II-2; (**c**) G-III-2.

An effective dynamic Brazilian splitting test must ensure the dynamic stress balance before failure, which can be verified by comparing the dynamic stress history at both ends of the specimen in the impact test. In each loading mode, the dynamic stress history of a typical specimen is shown in Figure 3. The dynamic stress on the incident side is the sum of incident stress and reflected stress, marked as Int+Re in the figure, and the dynamic stress on the transmitted side is transmitted stress. It can be seen from Figure 3a–c that before the peak stress, the dynamic stress had a good consistency at the incident and transmitted sides, which indicated that the specimen has reached the dynamic stress balance under the three loading modes, and tests were effective. The stress at both ends of the specimen became unbalanced due to the complete splitting failure of the specimen after the peak load.

### 3.2. Matching of the High-Speed Camera to Stress Loading Time

Although the high-speed camera and oscilloscope have achieved synchronous control, further processing is needed to make the stress loading time more accurately match with the images taken by the camera. The time when the stress wave propagates from the strain gauge on the incident bar to the contact interface between the specimen and the incident bar is denoted as *t_b_*. There is a time interval when the incident stress wave arrives at the strain gauge and the oscilloscope generates TTL electrical frequency signal and triggers the high-speed camera, and this time interval is denoted as *t_TTL_*. Therefore, the number of images collected by the high-speed camera when the specimen begins to be loaded by the incident stress wave is calculated as follows:

(8) $$n=\frac{t_{b}-t_{TTL}}{t_{\mathrm{frame}}},t_{b}=\frac{d}{C_{b}}$$

where *t*_frame_ is the time interval for the camera to capture images, namely 12.6 μs; *d* is the distance between the strain gauge and the incident bar end, which is 1.15 m.

**Figure 4:**
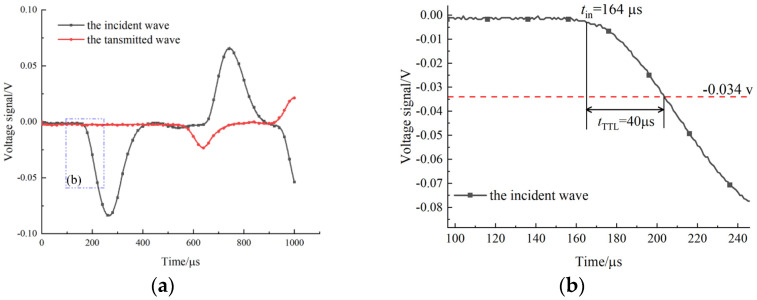
Original voltage signal of the specimen G-III-2 recorded by the oscilloscope (**a**); local magnification of the incident signal (**b**).

In addition, the TTL electrical frequency signal will be generated synchronously when the voltage signal monitored by the oscilloscope is smaller than −34 mv. Figure 4 shows the original voltage signal variation curve of the specimen G-III-2 during the whole loading process. It can be seen from the figure that *t_TTL_* is 40 μs. According to Equation (8), the value of *n* is 13.7, that is, the specimen has been subjected to about 3.8 μs of the impact load when the 14th image is captured by the high-speed camera. Therefore, the above analysis achieved the match between the stress loading of the specimen and the high-speed camera at the microsecond level, and the time of each image could be accurately determined.

### 3.3. Loading Rate of the Dynamic Brazilian Tests

**Figure 5:**
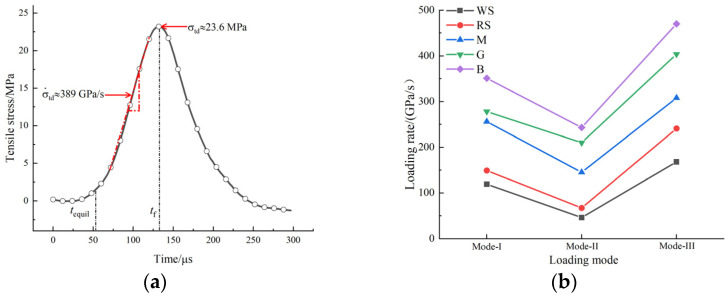
(**a**) History of tensile stress during loading of the specimen G-III-2; (**b**) the loading rate of each rock type under three loading modes.

In conventional dynamic compression tests, the mechanical properties and deformation parameters of materials are generally considered to be dependent on the strain rate. However, in the dynamic Brazilian test, a uniform value of strain rate cannot be obtained due to the uneven distribution of stress and strain in the disc specimen. Since the fixed incident energy is invariant in the dynamic Brazilian test, the loading rate was used to describe the loading condition of the disk in the Brazilian test [24]. The loading rate is determined by the slope of the straight-line segment before the peak value of the tensile stress–time curve in the center of the disc. Taking the specimen G-III-2 as an example, Figure 5a explains the method of determining the loading rate and tensile strength of the specimen. According to the figure, the loading rate and dynamic tensile strength are 389 GPa/s and 23.6 MPa, respectively. The loading rate of other specimens can be obtained according to the same treatment method, and all test data are sorted out and plotted in Figure 5b. It can be found that the loading modes have a significant influence on the loading rate of the specimen. For the same rock type, the loading rate is the highest under mode-I loading, which is the lowest under mode-II loading. In addition, due to the different natural properties of rock specimens, the loading rate of the specimen also shows significant differences.

### 3.4. Dynamic Tensile Strength and Deformation Characteristics of the Specimen

**Figure 6:**
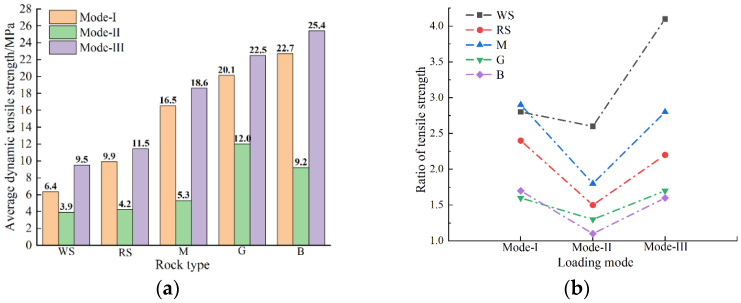
(**a**) Average dynamic tensile strength of different rock types under three loading modes; (**b**) the ratio of tensile strength of different rock types under three loading modes.

According to the test data, the dynamic tensile strength of all specimens was calculated by Equation (7). The average dynamic tensile strength of specimens of different lithologies under three loading modes is plotted in Figure 6 and Table 2. It can be seen from the figure that the loading mode has a significant impact on the dynamic tensile strength of the rocks, which is similar to the static test [31]. For all rock types, the dynamic tensile strength obtained by the mode-III loading is the highest, while the mode-II loading measures the lowest value. The dynamic tensile strength obtained by mode-I loading is similar to that of mode-III loading. However, there are also differences between the two tests (dynamic and static Brazilian tests) under different loading modes. First, the tensile strength of the specimen in the dynamic Brazilian test is higher than that in the static Brazilian tests. Second, the dynamic tensile strength obtained by mode-II loading is much lower than that of the other two loading modes. The Brazilian test under impact load seems to amplify the characteristics of these three loading methods, and the steel bar loading may greatly underestimate the dynamic tensile strength of the rock.

The dynamic tensile strength of the specimen was further compared with the static tensile strength, and the ratio of dynamic to static tensile strength was calculated, as shown in Figure 6b. Regardless of the loading modes, dynamic impacts can improve the tensile strength of rocks, and the increased strength of soft rocks (white and red sandstone) is more obvious. In addition, the increase ratio of tensile strength of all specimens under mode-III and mode-I loading are large and similar (except white sandstone), while the increase ratio under mode-II loading is the smallest.

**Figure 7:**
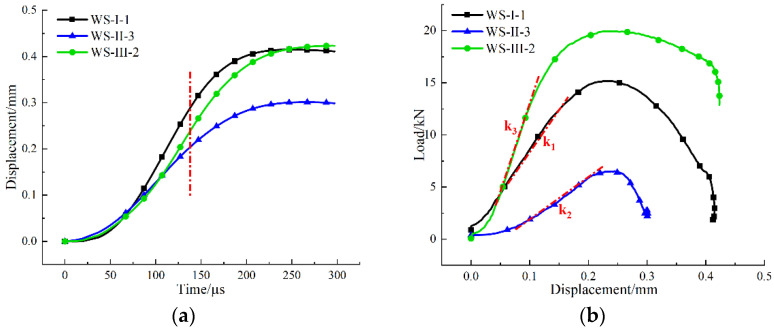
(**a**) Displacement–time curves and (**b**) loading–displacement curves of typical white sandstone specimens under three loading modes.

The displacement of the specimen at ends of the incident bar and the transmitted bar can be calculated by Equations (5) and (6). Since there was almost no displacement at the end of the transmitted bar when the specimen was subjected to impact load, only the displacement of the incident bar end was considered. Figure 7a shows the displacement–time curves of white sandstone specimens under three loading modes. The displacements of the specimens under mode-I and mode-III loading are large, while that of the white specimen under mode-II loading is the smallest. This is obviously different from the results of the static test [31], which is mainly because the stress concentration caused by mode-II loading is more intense under dynamic impact, and the steel bar almost has no time to penetrate the end of the specimen. In addition, a linear region was observed at the middle part of three displacement–time curves. In the linear region, the displacement values of the white sandstone specimens under the three loading modes were arranged in descending orders as mode-I, mode-III, and mode-II, respectively. The maximum displacement value of the specimen under mode-I loading was due to the shear breakage phenomenon at the end of incident bar, resulting in an increase in displacement compared with the specimen under mode-III loading. The minimum displacement value of the specimen under mode-II loading was because its loading rate was the lowest, that was, the increase of load per unit time was the least, resulting in the minimum displacement at the same time. A linear segment was observed at the loading–time curve and the displacement–time curve of the specimen, so a linear region should also appear at the load–displacement curve, as shown in Figure 7b. *k*_1_, *k*_2_, and *k*_3_ are the slopes of the straight section of the load–displacement curve of the specimen under the three corresponding loading modes, respectively. The slope can be regarded as the ratio of loading rate to displacement rate in the elastic deformation stage of the specimen under three loading modes, which shows similar characteristics to the load–displacement response of the specimen in the static Brazilian tests [31].

### 3.5. Dynamic Failure Analysis of Specimens

An effective dynamic Brazilian test was done to ensure that the specimen cracks at a certain point in the central region of the disc. Therefore, it is necessary to analyze the failure modes of the specimen under three loading modes. Since the high-speed camera was used in the dynamic Brazilian test, the deformation localization and fracture evolution process of the specimen could be captured more accurately than the industrial camera used in the static test [31]. Table 3 shows the full-filed tensile strain contour before the peak load of representative specimens under each loading mode.

It can be found from the table that the strain localization characteristics of the specimen in the dynamic Brazilian tests are highly similar to those in the static test, showing the same two distribution types. The arc loading mode under dynamic impact was still the most consistent with the Brazilian test theory. However, the strain distribution characteristics of the five rocks (except marble) under mode-I loading differed from the static tests. This may be because when the compressive incident wave propagated to the end of the specimen, relatively weak soft rock was prone to damage at the contact end, and then the damage was aggravated during the loading process. Hard rocks exhibited the opposite condition under transient impact and had sufficient time to reach stress equilibrium. In the following, dynamic failure analysis was carried out on typical specimens that conformed to the above two strain localization characteristics, and the failure process of other specimens was taken as a reference.

#### 3.5.1. Typical Fracture Characteristics I

**Figure 8:**
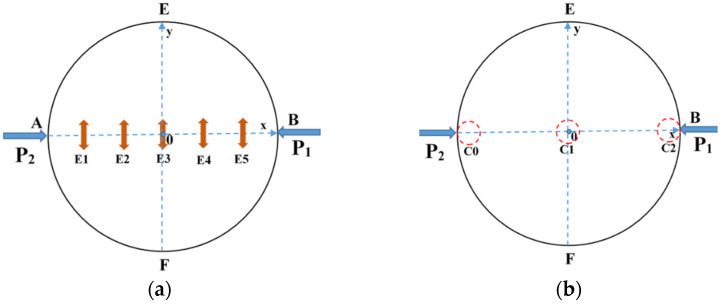
Arrangement of five virtual extensimeters (**a**) and three monitoring circles (**b**) on the specimen surface.

**Figure 9:**
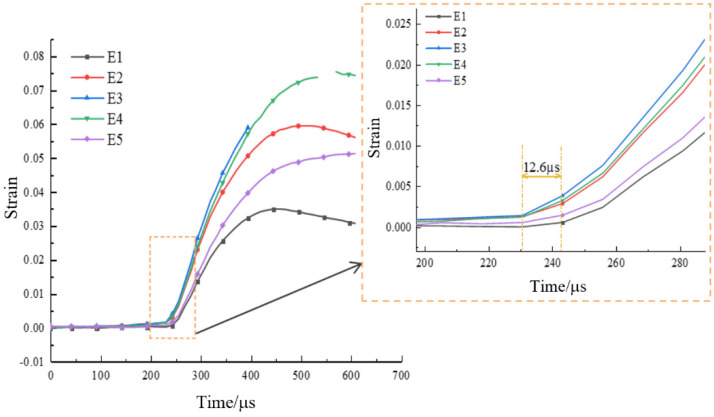
Strain history of five virtual extensimeters for the specimen WS-III-3.

A typical strain distribution characteristic under arc loading mode was selected for failure analysis. According to relevant theories in the static Brazilian tests, the tensile strain concentration in the center of the specimen can be considered as tensile stress concentration, and the disc specimen will crack from the center. The (The VIC-2D software version number is 6, and VIC-2D software invented by Correlated Solutions Inc. in Irmo, SC, USA) was used to arrange five virtual extensimeters (E1–E5) with equal spacing along the loading axis on the specimen surface (as shown in Figure 8a), and the tensile strain history (taking specimen WS-III-3 as an example) extracted by VIC-2D was plotted in the Figure 9. The tensile strain values of five extensimeters rose slowly, with tensile strain values below 0.25% before 230.6 μs. The increase rate of strain values of extensimeters E2, E3, and E4 started to accelerate, but that of E1 and E5 did not accelerate significantly. This also indicated that the region where the stress concentration occurred in the specimen was first in the center of the disc. After 12.6 μs (image interval), the strain increase rate of the five extensimeters rose sharply. However, the five strain extensimeters in descending order of strain values were E3, E4, E2, E1, and E5. The tensile strain value at the center of the disc was always the maximum. These data suggested that the dynamic crack propagation velocity was extremely rapid. Although the high-speed camera was used, the sequence of strain mutations of five extensimeters could not be accurately identified, and only the sequence of strain mutations of the regional extensimeters could be analyzed. However, the above analysis strongly indicates that the specimen must have started to crack at a point in the center of the disc, and then the fracture spread rapidly to both ends. The data from the virtual extensimeters showed that although the dynamic tensile strength of the specimens under arc loading and plate loading was not significantly different (the former was slightly greater than the latter), it was still necessary to use the arc loading method in the dynamic Brazilian test because it ensured, as much as possible, that the specimen would fracture from the center of the disc.

**Figure 10:**
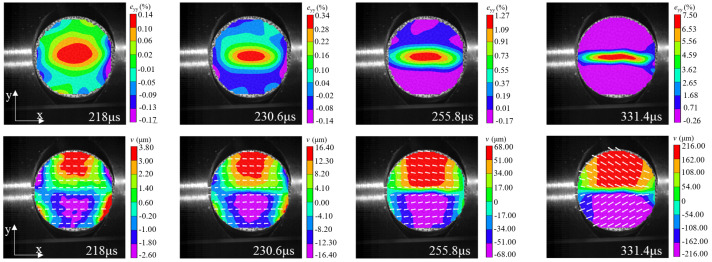
Strain and displacement contours of the specimen WS-III-3 at typical moments (impact load direction from right to left). The positive direction of the x-axis is the impact load direction, and the y-axis is perpendicular to the impact load direction.

In order to further analyze the deformation and rupture evolution of the specimen, the full-field displacement and strain contours at typical moments were extracted by VIC-2D, which is shown in Figure 10 (the top four images are strain contours and the bottom four images are displacement contours). The leftmost contour was the image of the specimen just before the peak, and the white arrow in the displacement contour represents the displacement vector. Before the peak load, the tensile strain was concentrated in the center of the disc, and the displacement contour showed a good symmetry without *y*-direction component of the displacement vector (the displacement in the *x*-direction was due to the compressive stress wave). When the time was 230.6 μs, the strain value increased significantly, while the surrounding strain value became smaller. In addition, the displacement vector in the center of the displacement contour started to have a slight *y*-direction component, indicating that the specimen was cracked at the center of the disc at this time. The moment of crack initiation point was in full agreement with the analysis by virtual extensimeters. After the crack initiation, the crack started to expand to both ends of the specimen, and the strain contour showed that the strain concentration zone spread to both ends with increasing of strain values. Meanwhile, the process of crack propagation could be well reflected indirectly from the displacement contour: firstly, the displacement concentration areas (red and purple areas) at the upper and lower ends of the specimen gradually spread to both ends with rising displacement values; secondly, the displacement vector in the center region of the disc became longer, and the displacement vector gradually tilted.

#### 3.5.2. Typical Fracture Characteristics II

**Figure 11:**
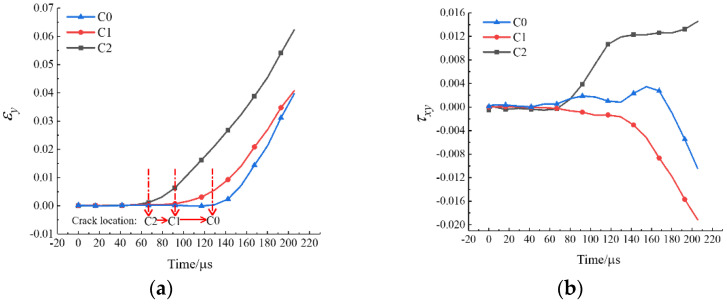
The *y*-direction strain (**a**) and shear strain (**b**) histories of three monitoring circles for the specimen WS-II-1.

The strain localization that occurred in the specimen under mode-II loading is another typical fracture characteristic. According to the relevant results in the static test analysis, it was known that for this loading case, the disc tended to have localized premature damage at the end. The following was an example of damage analysis for specimen WS-II-1. Three locations with high probability of crack initiation were marked in the Brazilian disc, as shown by the small red circles in Figure 8b, i.e., C0, C1, and C2. The diameter of these circles is about 5 mm, and their tensile and shear strains with time were extracted using VIC-2D, respectively, as shown in Figure 11. The strain at these three positions was almost unchanged until 66.8 μs. Then, the tensile and shear strains of C2 started to increase significantly, indicating that the shear crushing behavior occurred first due to the high stress concentration. A certain degree of shear stress was also present at the other end of the specimen, C0, but the stress value was small, and no damage occurred. After that, the same strain mutation occurred at C1 and C0, respectively, which indicated that the cracks at the end propagated rapidly to the left along the loading diameter after shear fragmentation occurred at the incident end of the disc. The clear damage sequence shows the superiority of the high-speed camera. In addition, it was worth noting that the moment of damage of the disc under mode-II loading (66.8 μs) was much earlier compared to that under mode-III loading (230.6 μs). The stress concentration at the end due to the steel bar loading method is undoubtedly more prominent under dynamic impact, which leads to lower slope of the load–displacement curve and tensile strength compared to the other two loading modes. Based on the above analysis, the mode-II loading was not suitable for determining the dynamic tensile strength of rock disc specimens.

**Figure 12:**
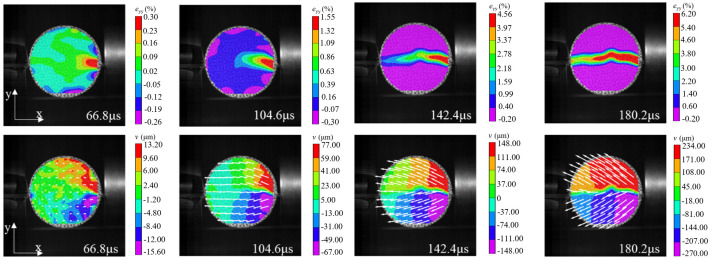
Strain and displacement contours of the specimen WS-II-1 at typical moments (impact load direction from right to left). The positive direction of the x-axis is the impact load direction, and the y-axis is perpendicular to the impact load direction.

The full-field strain and displacement contours at typical moments, as shown in Figure 12 (the top four images are strain contours and the bottom four images are displacement contours), were obtained by using the DIC technique to deeply analyze the deformation and rupture process of the disc under mode-II loading. The leftmost one was the image of the disc just before the peak load, and the white arrows in the displacement contour represent the displacement vectors. As can be seen from Figure 12, minor damage has occurred at the right end of the specimen due to the strain concentration before the peak load. The displacement vector at the right end of the specimen produced a vertical component, and the tensile strain and displacement values are larger here. After the peak load, the specimen underwent more obvious shear damage, and the macroscopic crack was formed at the end. The evolution of the strain contour at the top in Figure 12 reflected dynamic propagation process of the crack, and finally the specimen was completely split and damaged along the loading diameter. Similarly, the process of crack propagation from the incident end to the transmitted end can be well reflected from the displacement contour: firstly, the displacement concentration zone (red and purple areas) near the incident end of the specimen gradually propagated to the left and the displacement value became larger; secondly, the tilted displacement vectors in the right end of the disc gradually became longer and the displacement vectors at the left end gradually began to change from parallel to tilted.

### 3.6. Final Failure Pattern of Specimens

**Figure 13:**
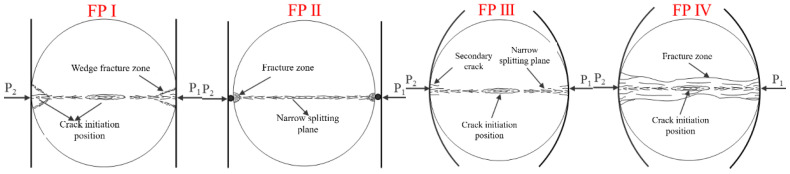
The four failure patterns of specimens in dynamic BD tests.

The final failure pattern of typical specimens for each loading method using images captured by the high-speed camera was shown in Table 4, and these specimens were consistent with those in Table 3. The final failure patterns of the researched specimens were summarized into four types by careful observation and combining the results of the analysis related to the DIC technique, as shown in Figure 13.

The failure pattern I (FP I) is a typical damage mode of the specimen under mode-I loading. Regardless of whether the specimen satisfied the central crack initiation, a certain degree of wedge-shaped fragmentation zone appeared at both ends of the specimen, and the central region along the loading diameter showed a narrower cleavage surface. The specimen was cracked at the center and the crack propagated to both ends of the disc causing complete splitting along the loading diameter. However, under the inertial impact of the incident bar, a wedge-shaped fragment would be formed at the end of the specimen due to shearing and twisting effects. The failure pattern II (FP II) is a typical damage mode of the specimen under the mode-II loading mode. Due to the dynamic impact and the strong stress concentration caused by steel bars, tiny crushing zones were generated at both ends of the specimen in contact with the steel bar, and ideal radial splitting was formed in the center of the disc specimen, with a relatively flat rupture surface and no excess secondary cracks generated. The failure pattern III and IV (FP III and IV) are two typical damage modes exhibited by specimens under mode-III loading. However, the FP III was only observed for white sandstone specimens, and the specimens were finally split and damaged radially from the center, accompanied by a very small amount of minor secondary cracks at the specimen ends. For rock specimens other than white sandstone, FP IV was observed. At higher loading rates, in addition to cracking damage along the center of the loading diameter, the specimen developed secondary cracks along the radial penetration at its two ends, thus eventually forming a fragmentation zone along the loading direction. The above analysis of failure modes again strongly suggested that dynamic loading further amplified the uniqueness of each loading method compared to static loading.

## 4. Conclusions

This paper mainly studied the damage law of dynamic Brazilian test under three loading modes. The relevant principles of the dynamic Brazilian test were introduced first, and the scheme and key operational steps of the test were discussed. The test results were compiled and analyzed in detail, including the tensile strength, deformation characteristics, and the final failure modes of the specimen. The main conclusions are as follows:

(1) The peak load and deformation characteristics of the dynamic Brazilian test were equally strongly influenced by the loading modes. The effects of loading modes on the tensile strength of the specimens were consistent with the static performance, i.e., the dynamic tensile strength determined by mode-III loading was the highest, while it determined that mode-II loading was lowest. In addition, the slope of the load–displacement curve of the specimen under mode-II loading was the smallest, while it was largest in mode-III loading.
(2) There were two typical fracture characteristics in the dynamic Brazilian test. The arc loading method showed outstanding superiority in the dynamic Brazilian test, and the results were more consistent with the Brazilian test theory compared with the other two loading methods. In contrast, the test results under the steel bar loading were unsatisfactory and may greatly underestimate the dynamic tensile strength of the rock.
(3) The specimens exhibited four different damage modes under three loading modes. In addition, it was incomplete or even wrong to collect only the broken pieces of specimens to determine the damage pattern and evaluate the damage mechanism of the specimen. On the contrary, the experiments can help scholars to better understand the evolution of rock fracture and damage mechanism by using the DIC technique.

## Figures and Tables

**Table 1:** Specimen dimensions and the dynamic Brazilian test results.

Specimens No.	*D*/mm	*t*/mm	*P_fd_*/kN	$\sigma_{td}$/MPa	Mean/MPa
WS-I-1	49.54	25.03	12.35	6.34	
WS-I-2	49.73	25.05	12.82	6.55	6.35
WS-I-3	49.32	25.04	11.95	6.16	
WS-II-1	49.12	25.03	7.36	3.81	
WS-II-2	49.16	25.08	8.25	4.26	3.91
WS-II-3	49.56	25.04	7.13	3.66	
WS-III-1	49.09	25.10	20.71	10.70	
WS-III-2	49.23	25.09	16.12	8.31	9.52
WS-III-3	49.62	25.06	18.67	9.56	
RS-I-1	49.10	25.02	16.96	8.79	
RS-I-2	49.34	25.11	17.83	9.16	9.88
RS-I-3	49.06	25.04	22.58	11.70	
RS-II-1	49.09	25.03	5.77	2.99	
RS-II-2	49.04	25.02	7.38	3.83	4.24
RS-II-3	49.02	25.03	11.39	5.91	
RS-III-1	49.45	24.98	22.12	11.40	
RS-III-2	49.13	25.01	24.70	12.80	11.53
RS-III-3	49.16	25.08	20.12	10.39	
M-I-1	49.14	25.06	33.46	17.30	
M-I-2	49.23	25.03	30.77	15.90	16.53
M-I-3	49.26	25.06	31.80	16.40	
M-II-1	49.25	25.04	11.35	5.86	
M-II-2	49.10	25.02	12.41	6.43	5.29
M-II-3	49.15	25.11	6.94	3.58	
M-III-1	49.28	25.13	44.16	22.70	
M-III-2	49.32	24.97	28.05	14.50	18.64
M-III-3	49.46	25.02	36.39	18.72	
G-I-1	49.41	25.06	43.18	22.20	
G-I-2	49.26	25.01	35.61	18.40	20.13
G-I-3	49.29	25.07	38.43	19.80	
G-II-1	49.39	25.04	23.19	11.94	
G-II-2	49.43	25.13	17.09	8.76	12.02
G-II-3	49.47	25.09	29.95	15.36	
G-III-1	49.19	25.14	40.40	20.80	
G-III-2	49.22	25.05	45.76	23.63	22.48
G-III-3	49.37	25.13	44.84	23.01	
B-I-1	49.45	25.11	40.57	20.80	
B-I-2	49.43	24.97	45.75	23.60	22.70
B-I-3	49.39	25.11	46.17	23.70	
B-II-1	49.51	25.01	18.61	9.57	
B-II-2	49.44	25.06	17.94	9.22	9.22
B-II-3	49.36	25.11	17.27	8.87	
B-III-1	49.51	25.07	55.21	28.32	
B-III-2	49.32	25.03	44.99	23.20	25.40
B-III-3	49.49	25.05	48.06	24.68	

**Table 2:** Test results of static uniaxial compression tests.

Rock Types	*σ_c_* (MPa)	*E* (GPa)	*ν*
White sandstone	37.7	9.2	0.15
Red sandstone	62.6	14.8	0.18
Marble	79.2	43.7	0.21
Granite	171.7	72.2	0.23
Basalt	158.4	61.9	0.25

**Table 3:** Contour of tensile strain before peak load of typical specimens under three loading modes.

Rock Type	White Sandstone	Red Sandstone	Marble	Granite	Basalt
Mode-I	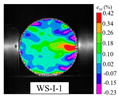	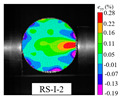	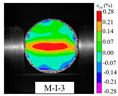	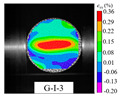	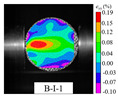
Mode-II	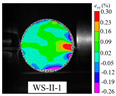	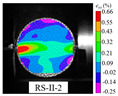	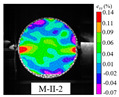	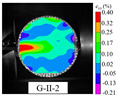	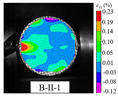
Mode-III	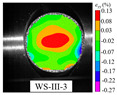	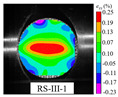	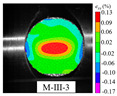	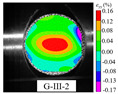	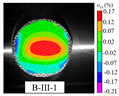

**Table 4:** The final failure patterns of typical specimens under three loading modes.

Rock Type	White Sandstone	Red Sandstone	Marble	Granite	Basalt
Mode-I	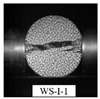	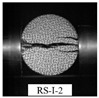	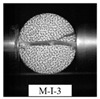	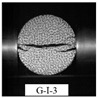	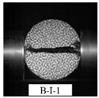
Mode-II	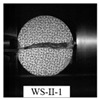	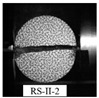	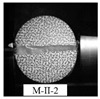	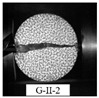	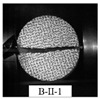
Mode-III	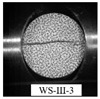	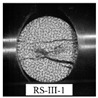	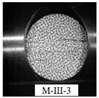	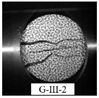	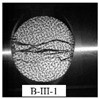

## Data Availability

The data presented in this study are available on request from the corresponding author.
